# Editorial Brevities

**Published:** 1887-02

**Authors:** 


### EDITORIALS AND MISCELLANEOUS.

### EDITORIAL BREVITIES.

MUMPS, measles, scarlet fever, and pneumonia are now *a la mode* in Atlanta.

A GOOD APPOINTMENT.—At a late meeting of the City Council Dr. W. S. Armstrong was re-elected a member of the Board of Health.

COCAINE, which originally sold at the rate of $4,220 per pound, is now reduced to two and a half cents per grain, or $12 per pound; and yet we are not happy!

THE FAITH CURE.—What is more pathetic (says the *Detroit Free Press*) than to see the simple faith with which a bald-headed man will buy an infallible hair restorer from a bald-headed barber?

THE title of Doctor (says the *Medical News*) was invented in the twelfth century at the first establishment of the Universities. William Gordenia was the first person upon whom the title of Doctor of Medicine was bestowed. He received it from the college at Asti in 1329.

PNEUMONIA.—A medical friend in Southern Alabama writes, requesting that our subscribers will write and publish in the RECORD their views in regard to the best treatment for pneumonia. We hope that a few, at least, will respond upon this interesting subject.

DEATH OF DR. J. M. BENTON.—We regret to learn of the death of Dr. J. M. Benton, of Beauregard, Miss. Dr. Benton was a young man—aged thirty-two years. He was a graduate of the University of Louisiana; was a good physician, and a useful member of the Methodist Church. He leaves an affectionate wife and two little children to mourn his demise. *Requiescat in pace.*

WELCOME.—With unfeigned pleasure we welcomed, this morning, to our sanctum the reappearance of "Squibb's Ephemeris." Owing to the absence from this country of the senior editor, the publication of this most valuable journal had been lately suspended. We congratulate Dr. Squibb upon his safe return, and the resumption of his arduous yet highly appreciated undertaking.

THE STEPHENS' PATENT SADDLE-BAG FOR PHYSICIANS.—In this issue of our journal we commence an interesting advertisement by George R. Hopkins & Co., wholesale Druggists, of St. Louis, Mo., who are the agents for the Stephens' Saddle-bags. The graduates of our colleges and physicians generally, who use the saddle-bag in practice, will do well to examine this bag, which is very highly recommended. They may be purchased in Atlanta, at the Instrument store of Mr. Isaac Phillips. Mr. Phillips is a very affable and pleasant business gentleman, and keeps an interesting and beautiful assortment of Surgical Instruments, etc., at No. 14 Marietta street.

SCOTT & BROWNE, manufacturing chemists, of New York (says the *Massachusetts Eclectic Medical Journal*), make a specialty of producing emulsion of cod-liver oil with hypophosphites. Their great care in selecting the oil and in making the combination is amply proven by the high therapeutical value set upon the emulsion by the profession. It is no new remedy, but has been steadily growing in demand for a number of years. It is certainly very useful in restoring wasting tissue, and in cases of scrofulous children it acts almost as a specific. They also offer a Buckthorn Cordial, which is highly useful in the treatment of constipation.

INTERNATIONAL MEDICAL CONGRESS.—There seems now to be no doubt but the International Congress to be held in Washington this coming Summer will prove a grand affair, and an honor to the medical profession of this country. We anticipate great results. We firmly believe that from the date of the opening of this Congress a new era will be introduced in the science and art of medicine, such as never has been witnessed before in the history of medical progress and achievements in any age. Every nationality will be duly represented in this great council. The brains of every clime will bring their mite to the urn of common lore, and carry back to their respective homes an impression no less fixed than enduring of the stupendous achievements which the medical mind of this country has wrought within the last decade.

