# Correction

**Published:** 1887-03

**Authors:** 


CORRECTION.—In our last we stated that our senior editor, Prof. Thomas S. Powell, had been appointed Vice-President of the Section on Gynæcology at the International Congress. We have since learned that his appointment is upon the Section of Obstetrics, which we regard as, perhaps, a greater honor, and more appropriate, than the other, as he is Professor of Obstetrics in the Southern Medical College of this city. W.

